# Exploring the Potential of Three‐Dimensional DNA Crystals in Nanotechnology: Design, Optimization, and Applications

**DOI:** 10.1002/advs.202302021

**Published:** 2023-06-16

**Authors:** Huating Kong, Bo Sun, Feng Yu, Qisheng Wang, Kai Xia, Dawei Jiang

**Affiliations:** ^1^ Shanghai Synchrotron Radiation Facility Shanghai Advanced Research Institute Chinese Academy of Sciences Shanghai 201204 China; ^2^ Shanghai Frontier Innovation Research Institute Shanghai 201108 China; ^3^ Shanghai Stomatological Hospital Fudan University Shanghai 200031 China; ^4^ Wuhan Union Hospital Tongji Medical College Huazhong University of Science and Technology Wuhan 430022 China; ^5^ Hubei Key Laboratory of Molecular Imaging Wuhan 430022 China; ^6^ Key Laboratory of Biological Targeted Therapy the Ministry of Education Wuhan 430022 China

**Keywords:** DNA crystallization, DNA nanotechnology, framework nucleic acid, nucleic acid crystallography

## Abstract

DNA has been used as a robust material for the building of a variety of nanoscale structures and devices owing to its unique properties. Structural DNA nanotechnology has reported a wide range of applications including computing, photonics, synthetic biology, biosensing, bioimaging, and therapeutic delivery, among others. Nevertheless, the foundational goal of structural DNA nanotechnology is exploiting DNA molecules to build three‐dimensional crystals as periodic molecular scaffolds to precisely align, obtain, or collect desired guest molecules. Over the past 30 years, a series of 3D DNA crystals have been rationally designed and developed. This review aims to showcase various 3D DNA crystals, their design, optimization, applications, and the crystallization conditions utilized. Additionally, the history of nucleic acid crystallography and potential future directions for 3D DNA crystals in the era of nanotechnology are discussed.

## Graphical abstract

The design and construction of three‐dimensional DNA crystals have been a significant focus of structural DNA nanotechnology, with a promising future to orient and position guest molecules to overcome bottlenecks in macromolecular crystallization and enable rapid structure determination. As the field continues to evolve, 3D DNA crystals are poised to play an increasingly important role in nanotechnology and nanomedicine.

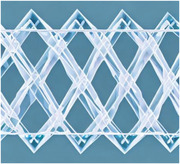

## 1 Introduction

**Figure 1:**
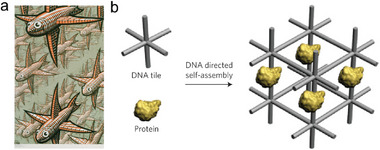
(Seeman's original proposal consisted of building 3D DNA scaffolds that could be used to orient proteins. a) M.C. Escher's “Depth”, Copyright 2017, The M.C. Escher Company‐The Netherlands, All Rights Reserved. The fish in this image is analogous to 6‐arm junctions. b) The prototype of DNA nanotechnology as outlined by Ned Seeman: using self‐assembled 3D DNA crystals to organize proteins, which would simplify the crystallographic study by eliminating the difficult process of growing crystals with high quality and determining their molecular structures by X‐ray diffraction. Reproduced with permission.^[^ ^9^ ^]^ Copyright 2011, Springer Nature.

DNA is well known as the carrier of genetic information in living organisms. Guided by the Watson–Crick base pairing rules, complementary oligonucleotides can recognize each other in complex physiochemical and biological environments, acting as a nature‐given tool of “Click” chemistry.^[^ ^1^ ^]^ Versatile nanoscale DNA structures and devices can be created by maneuvering DNA sequences in a programmable and predictable manner.^[^ ^2^, ^3^, ^4^, ^5^, ^6^, ^7^ ^]^ Inspired by the M.C. Escher woodcut Depth in which flying fish are arranged in a 3D array and the similarity between the rocket­like fish with horizontal and vertical fins and DNA 6‐armed junctions/tiles (**Figure** **1**a), Nadrian (Ned) C. Seeman first proposed rational design and assembly of three‐dimensional DNA crystals as periodic molecular scaffolds to align guest molecules that otherwise might be difficult to crystallize on their own (Figure 1b).^[^ ^8^, ^9^ ^]^ This has been proposed as one of the fundamental goals of DNA nanotechnology, to determine molecular structures while preventing unnecessary aggregation and promoting crystallization. 3D DNA crystals are also envisioned to benefit information storage, nanoelectronics, photonics, molecular sieves, and catalytic devices.^[^ ^9^, ^10^, ^11^ ^]^

**Figure 2:**
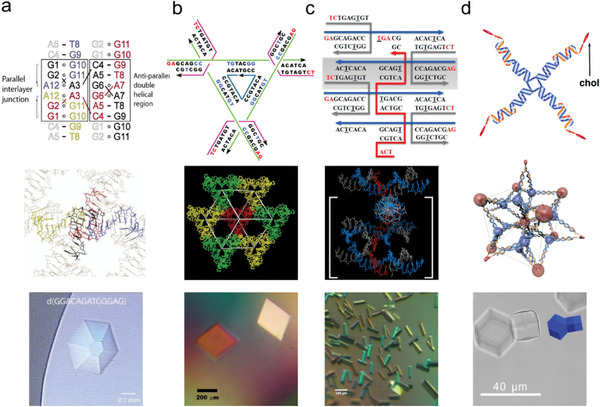
Four types of 3D DNA crystals. a) The DNA 13‐mer crystal. Reproduced with permission.^[^ ^12^ ^]^ Copyright 2004, Elsevier. b) The tensegrity triangle DNA crystal. Reproduced with permission.^[^ ^13^ ^]^ Copyright 2009, Springer Nature. c) The 4 × 5 structure DNA crystal. Reproduced with permission.^[^ ^14^ ^]^ Copyright 2016, American Chemical Society. d) The amphiphilic DNA C‐star crystals. Reproduced with permission.^[^ ^15^ ^]^ Copyright 2017, American Chemical Society. First row: Secondary structures of the motifs assembled into 3D DNA crystals. Middle row: Crystal structures of 3D DNA crystals. The diffraction resolution of the amphiphilic DNA C‐Star crystals was too low to determine the crystal structure. Therefore, the schematic of the arrangement of the self‐assembled DNA C‐Star is shown. Lower row: Optical images of 3D DNA crystals. Scale bars are shown in the figures.

Designing and building branching structures that are sufficiently rigid and uniform in three dimensions presents the main challenge in creating 3D DNA crystals. In 2004, Paukstelis and coworkers serendipitously developed a continuous 3D DNA crystal by self‐assembling a 13‐nucleotide DNA molecule, d(GGACAGATGGGAG) (**Figure** **2**a), in an experiment constructing lattices utilizing DNA and proteins.^[^ ^12^ ^]^ The DNA 13‐mer crystallized independently through self‐pairing interactions during the construction of a periodic DNA/protein lattice made from DNA four‐way junctions and tetrameric streptavidin, despite the existence of areas that are entirely complementary to its partner strands. They resolved the 2.1 Å crystal structure of DNA 13‐mer and found that DNA 13‐mer self‐assembled into a 3D lattice through base‐pairing and base‐stacking interactions. They described a method for building 3D DNA crystals that did not rely on Watson–Crick interactions. Despite the incomplete design of such DNA crystals, it provided a unique perspective on alternatives to Watson‐Crick base pairing in DNA self‐assembly and fortunately provided a method for creating porous three‐dimensional DNA lattices based on predictable non‐Watson‐Crick base pairs.

In 2009, Seeman, Mao, and coworkers developed the first 3D DNA crystal self‐assembled entirely based on Watson–Crick base pairing (Figure 2b).^[^ ^13^ ^]^ The crystal contains a single threefold symmetric DNA structural motif: tensegrity triangle. The motif features a linear extending along each triangle edge. As a result, the tensegrity triangle could grow into a periodic lattice by end‐to‐end replication of the pattern. The tensegrity triangle motif formed macroscopic (≈250 µm in size) rhombohedron‐shaped crystals that diffracted to 4.0 Å resolution as it was self‐assembled through the sticky ends of each DNA double helix. Some molecular details such as helicity and sugar pucker were revealed. The ingenious design and assembly of the tensegrity triangle crystals served as an inspiration for the creation of other types of DNA lattices.

In 2016, Yan, Seeman, and coworkers proposed that, by varying the inter‐crossover spacing and number of edges, DNA crystals can be programmed using a square motif resembling the tensegrity triangle (Figure 2c). This square motif is composed of a central 20mer oligonucleotide containing four self‐repeating sequences (red strand in Figure 2c).^[^ ^14^ ^]^ Four additional flank strands bind to the repeating sequence to form the corners of the central square motif. This motif has two‐nucleotide sticky‐ends on each arm. However, results of the modeling assay showed that the central strand was not sufficient to form a square, but rather formed a series of duplexes joined together by the central sequence. As such, they used sequences containing four and six identical repeats of five bases (4 × 5 and 6 × 5) as the central strand to assemble the crystals and determined their X‐ray crystal structures. The findings demonstrated that the lattice‐building process is only mediated by the repeated central unit, leading to the same topology for the 4 × 5 and 6 × 5 motifs. They developed a new technique for creating highly compact and ordered DNA lattices by employing various quantities of repeating sequences. On the basis of the layered arrangement of DNA helices, they subsequently created 3‐ and 6–fold symmetry‐structured DNA crystals. While base pairing provides fine control over the strength and selectivity of the interactions, its rigid “lock‐and‐key” nature means that higher‐order self‐assembly of programmed nanostructures requires precise geometric and thermodynamic optimization. This explains why self‐assembling 3D DNA crystals with a rational design is so uncommon.

In 2017, Di Michele and co‐workers constructed amphiphilic DNA motif “C‐stars” using cholesterol‐modified single‐stranded DNA (Figure 2d).^[^ ^15^ ^]^ Integrating the design flexibility and ease of functionalization of oligonucleotides, slow annealing of four core strands and four cholesterol‐modified end strands results in the formation of the C‐star, which further self‐assembles into single crystals (>40 µm) with lattice parameters greater than 20 nm. The advantage of this crystallization technique is that the process is possible through different interaction mechanisms between multivalent mediated C‐stars motifs by hydrophobic forces rather than Watson–Crick base pairing, thus eliminating the need for precise design and tight control of the DNA motif geometry, finally removing the design constraints that hindered the diversification of all DNA crystals. However, due to the nonspecificity of cholesterol interactions and intrinsic flexibility of C‐star motif, the diffraction resolution of this crystal can only reach 40 Å.

In this article, we scrutinize and delve into 3D DNA crystals, focusing in particular on the structure construction, optimization, and application of these 3D DNA crystals. We discuss the history of nucleic acid crystallography, review the crystallization conditions of DNA crystals, and survey how different factors (such as the temperature, ions, and crystallization methods) play their roles in forming the final products. In addition, we offer our perspectives on outstanding challenges of the design and development of 3D DNA crystals.

## 2 DNA 13‐mer Crystal and its Derivatives

**Figure 3:**
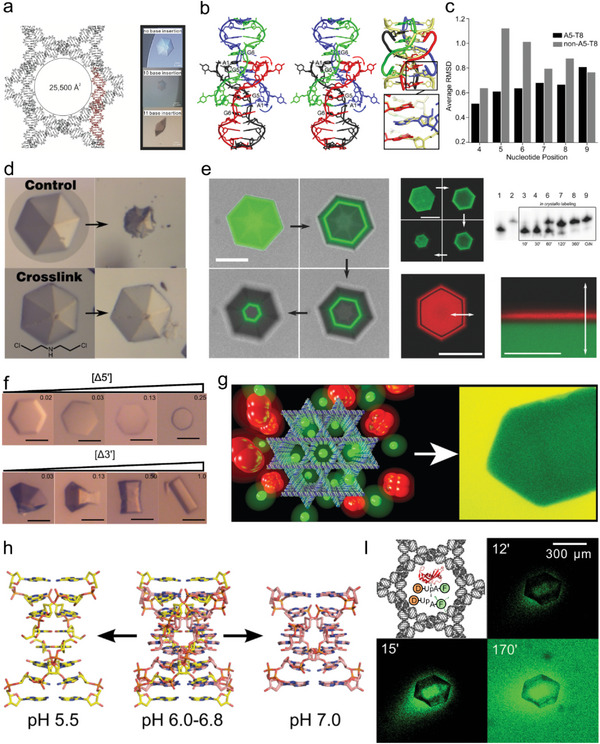
The DNA 13‐mer crystal and its derived crystals. a) The modeling and crystal images of the expanded “DNA 13‐mer” lattice. Reproduced with permission.^[^ ^12^ ^]^ Copyright 2004, Elsevier. b) An intercalation‐locked tetraplex. A1–A1 base pairs are intercalated between G5–G5 and G6–G6 pairs of opposite duplexes, locking the two duplexes into a tetraplex. Reproduced with permission.^[^ ^16^ ^]^ Copyright 2015, Oxford University Press. c) Average RMSD data of the duplex region of A5‐T8 and the non A5‐T8 structures. The A5‐T8 base pair is important for anchoring local structures. Reproduced with permission.^[^ ^17^ ^]^ Copyright 2015, Wiley‐VCH. d) The bis(2‐chloroethyl) amine crosslinking treatment enhanced the stability of DNA 13‐mer crystals under a variety of conditions. Reproduced with permission.^[^ ^18^ ^]^ Copyright 2016, Wiley‐VCH. e) The core‐shell assembly and postcrystallization labeling of DNA 13‐mer crystal. Reproduced with permission.^[^ ^19^ ^]^ Copyright 2017, Wiley‐VCH. (f) Tabular, acicular, and columnar habit modification of DNA 13‐mer crystals with increasing concentration of “poison” oligonucleotides. Reproduced with permission.^[^ ^20^ ^]^ Copyright 2017, American Chemical Society. g) DNA 13‐mer crystals as molecular sieves for selectively adsorbing proteins based on size. Reproduced with permission.^[^ ^21^ ^]^ Copyright 2006, American Chemical Society. h) pH‐induced structural transition in a single crystal. Reproduced with permission.^[^ ^22^ ^]^ Copyright 2012, American Chemical Society. i) RNase A‐infused expanded DNA 13‐mer crystals as solid‐state catalysts for cleavage of RNA substrate. Reproduced with permission.^[^ ^23^ ^]^ Copyright 2014, American Chemical Society.

The DNA 13‐mer crystal is a continuous hydrogen‐bonded 3D DNA consisting primarily of non‐Watson–Crick base pairs. It consists of stacked parallel helix layers, with adjacent layers connected by parallel strand base pairing. The 3' end of each DNA 13‐mer is oriented into a solvent channel which is large enough to allow 3' attached guest molecules to enter the crystal. Paukstelis and coworkers created a full turn of the antiparallel helix by inserting 10–11 bases between the helical region and the interlayer linkage and using a second complementary strand (**Figure** **3**a).^[^ ^12^ ^]^ This resulted in 356,000 Å^3^ expanded solvent space per unit cell in the channels. The modeling results showed that the significantly extended solvent channel in the lattice could accommodate a 45 kDa fungal mitochondrial tyrosyl‐tRNA synthetase and thus serve as a macromolecular scaffold for the protein.

### 2.1 Development

In 2015, Paukstelis et al constructed the first 3D DNA crystal (DNA 11‐mer) containing four pairs of different symmetric homo base pairs (Figure 3b).^[^ ^16^ ^]^ Two of these parallel duplexes are co‐axially stacked in opposite directions and are located between adjacent G‐G base pairs via intercalation of the 5’‐most A‐A base pairs. The intercalation region is a novel DNA tertiary structure motif similar to the i‐motif. Similar crystals can be produced by replacing the internal d(GAT) sequence with d(GAC). As a reasonable extension of the above‐mentioned observations, the ps d(GAY)‐containing duplexes may thus serve as a helpful motif for creating DNA nanostructures and crystals. Then they looked into how the Warson–Crick hybridization area affected the DNA 13‐mer crystal in more details.^[^ ^17^ ^]^ The findings demonstrate that a particular base pair (A5‐T8) has an impact on the propensity for crystallization and the self‐assembly rate of crystals. The hexametric duplex region may adopt a more favorable overall structure for crystallization by acting as a structural anchor during crystal assembly thanks to the A5‐T8 base pair (Figure 3c). This study also showed the capability of numerous novel DNA oligonucleotide sequences to adopt a homogeneous crystal structure. Those crystals were less thermally stable and needed a higher cation concentration to maintain integrity mainly because they had shorter Warson‐Crick hybridization zones. In 2016, they demonstrated a simple method to improve the stability of DNA crystals.^[^ ^18^ ^]^ Zhang et al found that DNA alkylated bis(2‐chloroethyl) amines could form interstrand crosslinks in 13‐mer DNA crystals, whose high‐temperature stability, low cation concentration stability, DNase I resistance, and longevity in the tissue culture medium could be enhanced by this cross‐linking procedure while maintaining the crystal's X‐ray diffraction features (Figure 3d). In 2017, McNeil et al. revealed the first example of biomacromolecular core‐shell crystal growth and layer‐by‐layer functionalization.^[^ ^19^ ^]^ Additionally, they demonstrated for the first time that covalent postcrystallization modifications are possible through DNA crystal solvent channels by employing fluorescent dyes to show the assembly procedure (Figure 3e). In 2017, Zhang et al. have investigated techniques that can modify and control the formation of 3D DNA crystals by DNA 13‐mer self‐assembly (Figure 3f).^[^ ^20^ ^]^ They designed 5' and 3' truncated versions of oligonucleotides, namely “toxic” oligonucleotides, that can still form Watson–Crick base pairs with the full‐length strand but were defective in forming noncanonical base pairs. The introduction of “toxic” oligonucleotides specifically disrupts key noncanonical base repair interactions in the crystal lattice, which could tweak the shape of the crystal, thus led to predictable changes in crystal habit. The ability to manipulate the properties of nano‐ and macroscale crystals expanded the scope of DNA crystal design and facilitated the construction of macroscopic objects with controllable and exploitable shapes.

### 2.2 Application

In 2006, Paukstelis and coworkers used extended “DNA 13‐mer crystals” as molecular sieves and soaked DNA crystals with proteins to investigate their ability to accommodate proteins of different molecular weights (Figure 3g).^[^ ^21^ ^]^ After one week of immersion, the results showed that proteins with molecular weights below 45 kDa could enter the crystal, and the amount of protein entering the crystal was inversely proportional to its size. This is in agreement with previous molecular modeling experiments. In 2012, Muser et al developed a pH‐responsive crystal of DNA 19‐mer (Figure 3h).^[^ ^22^ ^]^ They created crystals with varying unit cell dimension along the quadruple symmetry axis by extending the self‐complementary B‐DNA hybridization area. All but one or two designed secondary structure interactions could be produced when crystals are grown at different pH conditions. Surprisingly, there were discrepancies in crystal stacking between the predicted and observed structures as a result of the conformational alterations in the intended Watson–Crick duplex area. The planned noncanonical motif was nearly identical to the template when the crystals were produced at pH 5.5. While they observed a previously unexpected C‐G‐G‐C quadruple base pair on this structurally changed motif at pH 7.0. This study revealed that the 5′‐CGAA motif was structurally modular in response to pH values. In 2014, Geng et al. wrapped RNase into an expanded “DNA 13‐mer crystal” and demonstrated that RNase wrapped in the solvent channel of the DNA crystal was catalytically active (Figure 3i).^[^ ^23^ ^]^ The enzyme inside the DNA crystal was a solid‐state biomolecular catalyst made completely of biomolecules, and its kinetic characteristics are similar with those of other immobilized enzymes. This study presented the potential to develop modular catalysts by incorporating different enzymes in the solvent channels of DNA crystals.

## 3 Tensegrity Triangle Crystal

The Watson–Crick pairings in the DNA 13‐mer crystal and its derivatives were not full rationally designed in all cohesive directions, making it impossible to use these systems to conveniently build an arbitrary number of components in asymmetric cells. The tensegrity triangle crystal, consisting of a three‐fold symmetric motif that self‐assembled into a crystal, is the first fully designed 3D DNA lattice. Its helical axis extends linearly independently over each triangle edge, thus defines the pattern. Three single DNA strands in a precise 3:3:1 ratio are necessary to create the motif. The central strand contains three repeats of seven bases that pair with three identical edge strands to form a central triple symmetric triangle with a single nick position. These pairings occur between the central 7 nt of the 21‐nt edge strand, leaving 7 free bases on each side. Three cross‐strands are added, forming a four‐armed branch connection at the triangle's apex, which holds these seven bases together and helps them to be oriented. The tensegrity triangle motifs are assembled through the two‐bases sticky ends on the 5' end of the cross‐strands and duplex strands, then forming larger higher‐order periodic structures: DNA crystals.^[^ ^13^ ^]^

### 3.1 Development

**Figure 4:**
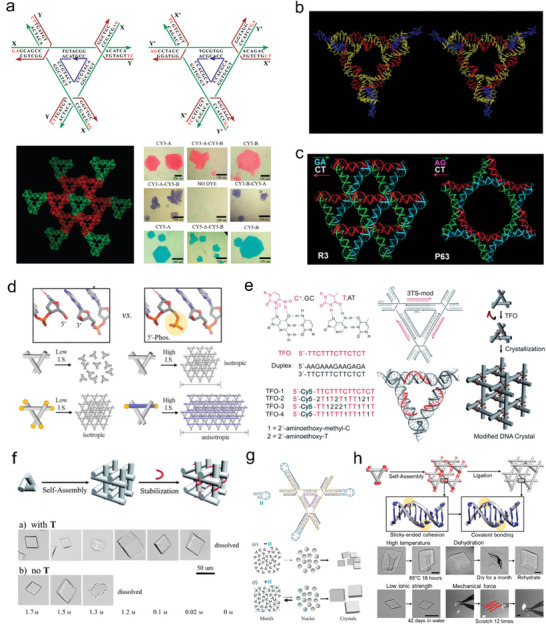
The development of tensegrity triangle crystals. a) The tensegrity triangle crystals are designed to contain two guest molecules per asymmetric unit. Covalent attachment of Cy3 and Cy5 to the independent motifs could produce crystals with different colors. Reproduced with permission.^[^ ^24^ ^]^ Copyright 2010, American Chemical Society. b) The crystal structure of the three‐turn triangle lacking a C at the crossover site in the crossover strand. The structure is identical to that of the tensegrity triangle crystals. Reproduced with permission.^[^ ^26^ ^]^ Copyright 2012, Wiley‐VCH. (c) The straight helices in the R3 model (PDB ID: 3GBI) and the bent helices in the P63 model (PDB ID: 7R96). Reproduced with permission.^[^ ^30^ ^]^ Copyright 2021, American Chemical Society. d) 5′‐phosphorylation promoted both tensegrity triangle crystal nucleation and growth along designed directions. Reproduced with permission.^[^ ^32^ ^]^ Copyright 2021, American Chemical Society. e) Strategy for the functionalization of tensegrity triangle crystals with TFO. Reproduced with permission.^[^ ^34^ ^]^ Copyright 2014, Wiley‐VCH. f) TFO functionalized tensegrity triangle crystals could be stable at ionic strengths as low as that of a 0.02 M solution of (NH_4_)_2_SO_4_. Reproduced with permission.^[^ ^35^ ^]^ Copyright 2015, Wiley‐VCH. g) Strategy for the modulation of assembly of tensegrity triangle crystals with the rationally designed 12‐nt‐long hairpin strand. Reproduced with permission.^[^ ^38^ ^]^ Copyright 2018, Wiley‐VCH. h) Postassembly enzymatically ligation of tensegrity triangle crystals enhanced the stability of the crystal in high temperature, dehydration, low ionic strength, and mechanical pulling. Reproduced with permission.^[^ ^40^ ^]^ Copyright 2019, American Chemical Society.

In 2010, Seeman and coworkers designed an asymmetric cell containing two tensegrity triangle molecules (**Figure** **4**a) ∖.^[^ ^24^ ^]^ In the asymmetric cell, each tensegrity triangle was only able to pair with the other and this pattern extended to form a crystal. The unit cell volume of such crystal is within 4.5% of exactly twice that contains only a single tensegrity triangle per unit cell. Additionally, it was demonstrated that the alteration in the crystals' coloring attributes was brought about by the covalent bonding of the dye molecules to the two various tensegrity triangles. Seeman's study established the feasibility of constructing species‐specific arrangements of macromolecular crystals in three dimensions using specific designs. In 2011, Timsit et al. suggested that tertiary interactions between phosphate and cytosine N(4) groups played an important role in the intermolecular cohesion in the tensegrity triangle crystal.^[^ ^25^ ^]^ To resolve this issue, Seeman et al. further demonstrated experimentally that tertiary interactions at the junctions involving cytosine N(4) proton were not responsible for the exits of tensegrity triangle structure, and that the formation of this crystal relied only on base pairing and covalent interactions (Figure 4b).^[^ ^26^ ^]^ Stahl et al. explored the role of heterogeneity and Watson−Crick base paring strengths in the tensegrity triangle crystal growth. They demonstrated that the crystal growth sensitively depended on the sequences of base pairs next to the Watson−Crick sticky ends.^[^ ^27^ ^]^ In 2017, Seeman et al. added additional base pairs to the tensegrity triangle motif, imposing significant torsional stress without affecting assembly and crystallization.^[^ ^28^ ^]^ In 2017, Conn et al built a time‐lapse microscope for monitoring of the growth process of tensegrity triangle crystals. This microscope improved our understanding of the crystal nucleation and growth process and helped to perfect the crystal growth settings by allowing direct observation of the crystal as a function of time and temperature.^[^ ^29^ ^]^ In 2021, Lu et al. used non‐canonical 5‘‐AG and 5’ ‐TC sticky ends in other isomorphic tensegrity triangles to complete crystal self‐assembly in the P63 hexagonal space group (Figure 4c).^[^ ^30^ ^]^ They observed that the non‐canonical interactions of the sticky ends had a profound effect on the local geometry of the crystal, changing the global topology during crystal self‐assembly. In 2012, Sha et al. discovered that the presence of a phosphate group on the 5’ end improved the resolution of tensegrity triangle crystals.^[^ ^31^ ^]^ In 2021, Li et al. found that the 5’ phosphorylation on the sticky ends not only promoted crystalline nucleation but also accelerated the growth of the crystal along the design direction (Figure 4d).^[^ ^32^ ^]^ In 2021, Li et al. investigated the fundamental mechanical properties and behavior of the tensegrity triangle crystals.^[^ ^33^ ^]^ Coarse‐grained molecular dynamics simulations were performed, and results were confirmed by nanoindentation experiments using atomic force microscopy. Various deformation modes were observed, including tension‐free, linear elasticity, double‐strand dissociation, and single‐strand component stretching. It was found that the mechanical properties of the tensegrity triangle structures are correlated with those of their components, but the structures exhibited complex behaviors that may not be predicted by single components.

The diffraction resolution and stability of the tensegrity triangle crystals are critical to be used as molecular hosts to resolve molecular structures. When the tensegrity triangle crystal was first developed in 2009, the resolution was only nearly 4 Å, which was far too small to accommodate guest molecules to perform structural analysis. Moreover, self‐assembled tensegrity triangle crystals were fragile due to their weak inter‐unit cohesion. Seeman, Mao, and coworkers performed a series of works to optimize the resolution and stability of the tensegrity triangle crystals. In the beginning, the predictable way to introduce guest molecules into tensegrity triangle crystals was to bind them to ssDNA and embed them in the crystal at high temperatures and go through slow annealing steps for assembly. However, most molecules are not able to withstand such harsh conditions. Moreover, the introduction of molecules may also interfere with the formation of crystals. On this issue, Seeman, Mao, and coworkers developed triplex‐forming oligonucleotides (TFOs) that can bind within the major groove of dsDNA by generating base triplets under mild acidic conditions (pH ≈ 5.0) after annealing (Figure 4e).^[^ ^34^ ^]^ This method allows the addition of guest molecules into intricate DNA patterns while overcoming the issue of preattaching a component to the DNA scaffold. In 2015, they introduced TFO to enhance the inter‐motif interactions, and greatly improve the stability of tensegrity triangle crystals (Figure 4f).^[^ ^35^ ^]^ Original tensegrity triangle crystals were stable only in solutions of high ionic strength, such as (NH_4_)_2_SO_4_ (1.2 mM). In contrast, TFO‐enhanced tensegrity triangle crystals could be stable in solutions with ionic strengths as low as 0.02 mM of (NH_4_)_2_SO_4_. Furthermore, photo‐crosslinking techniques based on TFO design have been developed.^[^ ^36^ ^]^ The introduction of TFOs could guide the photo‐crosslinking embedding of 4,5',8‐trimethylpsoralen at fingertip sites inside the crystal, thus improving the thermal stability of the crystal. Recently, Zhao et al. used the strand displacement of TFO in tensegrity triangle crystals by coloring the crystals with covalently attached fluorescent dyes. During the process of displacing the TFOs, the stability and integrity of the tensegrity triangle crystals were not substantially affected.^[^ ^37^ ^]^

Having developed stable tensegrity triangle crystals, regulating crystal resolution remain another goal to persue for biomacromolecule analysis. In 2018, Mao, Seeman and co‐workers introduced a 12‐nt‐long hairpin chain (H strand), which contained a 2‐nt sticky end complementary to the three ends of the tensegrity triangle (Figure 4g)^[^ ^38^ ^]^ The H strand could instantaneously bond to the surface of growing tensegrity triangle motif and tensegrity triangle crystals, reducing the number of crystal nuclei and the crystal growth speed, thereby producing larger crystals with slightly higher resolution. In 2018, Ohayon et al. analyzed the effects of lengths and sequences of sticky ends, as well as 5'‐ and 3' – phosphate on the formation and resolution of tensegrity triangle crystals.^[^ ^39^ ^]^ The results show that the crystal resolution for a 1‐nt sticky end (G:C) and a 3‐nt sticky end (GAT: ATC) were 3.4 and 4.2 Å, respectively. The position of 5' phosphate on the crossover (1), helical (2) or central strand (3) affected the resolution of the tensegrity triangle crystals for the 2‐turn monomer (3.0 Å) and 2‐turn dimer sticky ended (4.1 Å) systems. 3'‐ phosphate modification could also improve the crystal resolution. In 2019, they developed a method of crystal reinforcement after assembly, using DNA ligase to enzymatically connect the sticky end junction to make it covalent and thus improve the crystal stability (Figure 4h).^[^ ^40^ ^]^ Enzymatically ligated tensegrity triangle crystals could withstand high temperature, dehydration, low ionic strength and mechanical pulling. Moreover, the enhanced tensegrity triangle crystals could function effectively in biocatalysis and protein capture.

### 3.2 Applications

**Figure 5:**
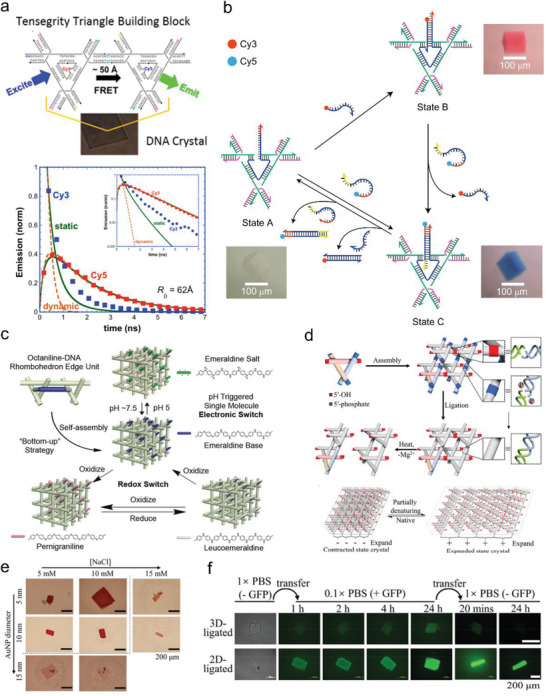
The applications of tensegrity triangle crystals. a) Fluorescence and energy transfer in dye‐labeled DNA crystals assembled by two‐triangle tensegrity motifs. R_0_(Cy3‐Cy5) is 62 Å. Reproduced with permission.^[^ ^41^ ^]^ Copyright 2016, American Chemical Society. b) Operation of the three‐state color tensegrity triangle crystal based on addition of a dye‐containing strand or a strand complementary to the dye‐containing strand. Reproduced with permission.^[^ ^42^ ^]^ Copyright 2017, Springer Nature. c) Octaniline attached tensegrity triangle motifs self‐assembled into a 3D crystal with four different switchable electronic states under mild conditions. Reproduced with permission.^[^ ^43^ ^]^ Copyright 2017, Wiley‐VCH. d) Design of stimuli‐responsive enzymatically ligated tensegrity triangle crystals. e) AuNP‐encapsulation behavior of the tensegrity triangle crystals at different NaCl concentrations. f) Encapsulation His‐tagged green fluorescence protein–ubiquitin deamidase (GFP‐DA) fusion protein in the tensegrity triangle crystals. Reproduced with permission.^[^ ^44^ ^]^ Copyright 2022, Wiley‐VCH.

A number of applications based on the tensegrity triangle crystals have been developed. In 2016, Melinger et al. investigated the fluorescence and energy transfer properties of tensegrity triangle crystals self‐assembled by Cy3 and Cy5 dyes modified tensegrity motifs based on an “asymmetric” design (**Figure** **5**a).^[^ ^41^ ^]^ The dyes attached in the crystals were in a more homogeneous environment compared to the reference dyes‐labeled DNA strands in solution, suppressing excited‐state isomerization and leading to a 2‐fold increase in the fluorescence intensity of Cy3 and a single‐exponential decay of Cy3 fluorescence. Moreover, a fluorescence resonance energy transfer (FRET) effect occurred in the tensegrity triangle crystals. This study demonstrated the ability of tensegrity triangle crystals to manipulate the placement, density, attachment chemistry, and orientation of dyes for future photonic applications. Furthermore, in 2017, Seeman and coworkers developed a three‐state DNA device that allowed the tensegrity triangle crystal to achieve three color transitions through dyer‐labeled chain displacement reactions (Figure 5b).^[^ ^42^ ^]^ This is the first report on the development of a stand‐alone device in 3D crystals. One of the ultimate goals of DNA nanotechnology is to organize nano‐electronic components into 3D arrays. The construction of tensegrity triangle crystals provided a unique “bottom‐up” strategy for 3D assembly of semiconductor molecules. In 2017, Seeman, Canary, and coworkers assembled 3D arrays of organic semiconductors using the tensegrity triangle motif (Figure 5c) .^[^ ^43^ ^]^ An organic semiconductor molecule: octamethyl laniline (octane) was integrated into a tensegrity triangle dimer, then self‐assembled to form a 3D crystal. As observed by color changes and Raman microscopy, the pernigraniline and leucoemeraldine states are retained between the reversible redox conversion of aniline inside this crystal. At pH 5, proton doping occurred to obtain the ancemeraldine salt state of aniline, corresponding to the conductive form of polyaniline. Octane could shift states quite freely in tensegrity triangle crystals, indicating the viability of this semiconductor device. Recently, Zheng et al. found that adhesion‐end cohesion could be reversibly formed or dissociated when exposed to external stimuli such as pH, temperature, and redox reagents, leading to reversible expansion and contraction of tensegrity triangle crystals along the non‐linked direction, within a range of 50 µm (Figure 5d).^[^ ^44^ ^]^ During this process, the porosity (i.e., permeability) of the crystals changed considerably. This expansion and contraction also provided a unique mechanism to control the encapsulation and release of guests, such as gold nanoparticles (AuNPs) and proteins (Figure 5e,f).

## 4 DNA Crystal Based on DNA Helices Layers

Compared to the tensegrity triangle motif, the multilayer motif guided by a central strand containing repetitive sequences required a decrease in the number of crossover bases from 7 to 5, where there is now a total of 4 repetitive sequences instead of 3 (4 × 5 nt). Therefore, a slight underwinding with respect to the two complete turns occurred in the motif, because 4 × 5 bp < 3 × 7 bp (21 bp = 2 × 10.5 bp). Instead of the anticipated “tensegrity square” crystal structure, the motif is assembled into a series of 21 base pair multilayers linked together by a central strand containing repetitive sequences, producing four attached duplex “blocks”.^[^ ^14^ ^]^ The dense arrangement of successive helical layers in the crystals lacked the uniform periodicity of the void space required in the initial design, and the cavities were too small for the accommodation of the guest molecules. Yan and coworkers therefore made further modifications to the motif to produce a more uniform arrangement of cavities.

**Figure 6:**
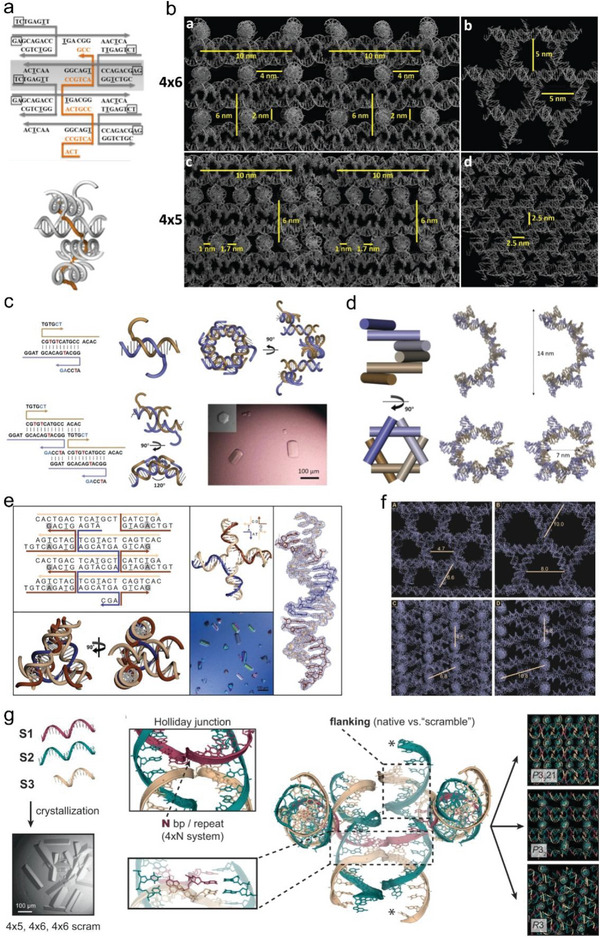
DNA crystal based on DNA helices layers. a) The secondary structure and 3D cartoon representation of the 4 × 6 motif. A central strand containing 4 repeats of a 6‐base sequence (4 ×6) connects each of four 21‐bp duplexes. b) Stereo view of the crystal packing of 4 × 6 and 4 × 5 motifs. In contrast to the 4 × 5 motif crystal, the 4 × 6 motif crystals contained a much larger cavity. Reproduced with permission.^[^ ^45^ ^]^ Copyright 2017, American Chemical Society. c) The secondary structure, 3D modeling, and crystal images of the self‐assembled hexagonal prism DNA crystals based on the duplex motif. d) 3D modeling and stereo view of the structure of DNA crystal with a layered hexagonal lattice. Reproduced with permission.^[^ ^46^ ^]^ Copyright 2018, Wiley‐VCH. (e) The secondary structure, 3D modeling, and crystal images of the 4 × 7 motif. A central strand containing 4 repeats of a 7‐base sequence (4 × 6) connects each of four 21‐bp duplexes. f) Stereo view of the crystal packing of 4 × 7 motifs with expanded crystal cavities by “3‐turn” design. Reproduced with permission.^[^ ^47^ ^]^ Copyright 2020, Wiley‐VCH. g) The composition of the Holliday junction is vital for the self‐assembly of 3D DNA lattices. Reproduced with permission.^[^ ^48^ ^]^ Copyright 2022, Springer Nature.

In 2017, Simmons et al. redesigned the “tensegrity square” and named it 4 × 6 motif (**Figure** **6**a).^[^ ^45^ ^]^ There was an extra base on each repeated sequence in the central strand of the motif. This modification reduced the structural strain caused by a single base defect in the central strand and prevented the extra filling required for the strictly periodically aligned cavities throughout the crystal. 4 × 6 motif crystals contained a 4 × 2 × 5 nm cavity, which is much larger compared to the cavity within the 4 × 5 motif crystal (Figure 6b). In addition, they assembled fully nuclease‐resistant mirror image crystals (L‐DNA) with the same sequence, allowing the encapsulation and protection of the guest molecule in a strictly nuclease‐free environment. In 2018, Zhang et al. rationally designed and clarified the first 3D DNA crystal with a layered hexagonal lattice (P6), in which two 21‐base oligonucleotides are employed to form a bimodal body, which is further assembled into a repeating array of Holliday junctions (Figure 6c).^[^ ^46^ ^]^ Unlike previous three‐layer crystals, there is no central strand to hold the layers together. Within the crystal, all interactions are based strictly on programmable Warson–Crick base pairing. The relatively large (≈450 nm^3^) cavities of this six‐fold symmetric lattice could accommodate larger guest molecules. A single fixed Holliday sequence (named “J1”) was used in all of the above crystals (Figure 6d). In 2020, Simmons et al. used a unique junction sequence (“J10”) with 4 repeats of a 7‐base sequence within the central strand (4 × 7 motif) and produced a significant increase in resolution to 2.7 Å, furtherly improved to 2.6 Å using a TA sticky end (Figure 6e).^[^ ^47^ ^]^ Larger pores and cavities were produced within the crystal (10 nm diameter), while maintaining crystal symmetry, for potential site‐specific localization of guest molecules (Figure 6f).

Recently, Simmons et al. investigated the effect of holiday junction sequences and dynamics on the self‐assembly of DNA crystals (Figure 6g).^[^ ^48^ ^]^ 36 fixed Holliday junction sequences were selected, and the effect of Holliday junction sequences on self‐assembled DNA crystals was studied using X‐ray crystallography. The results showed that most Holliday junction sequences could mediate the production of crystals, some of which enhanced the diffraction resolution or led to unique crystal symmetries. Unexpectedly, the sequences besides the Holliday junction sequences affect the crystal assembly significantly. Six of the Holliday junction sequences completely resisted being crystallized. Molecular dynamics simulations showed that these Holliday junction sequences were in lack of always lacked two discrete ion‐binding sites, which proved essential for crystal assembly. They screened all 36 Holliday junction sequences and identified consistently fatal sequence for the crystal formation. They systemically introduced the effect of different Holliday junction sequences, flanking sequence modifications, and the ability of the Holliday junction to trap ions on the rational design of self‐assembling DNA motifs assembled to form crystals.

## 5 Amphiphilic DNA C‐Star Crystal

**Figure 7:**
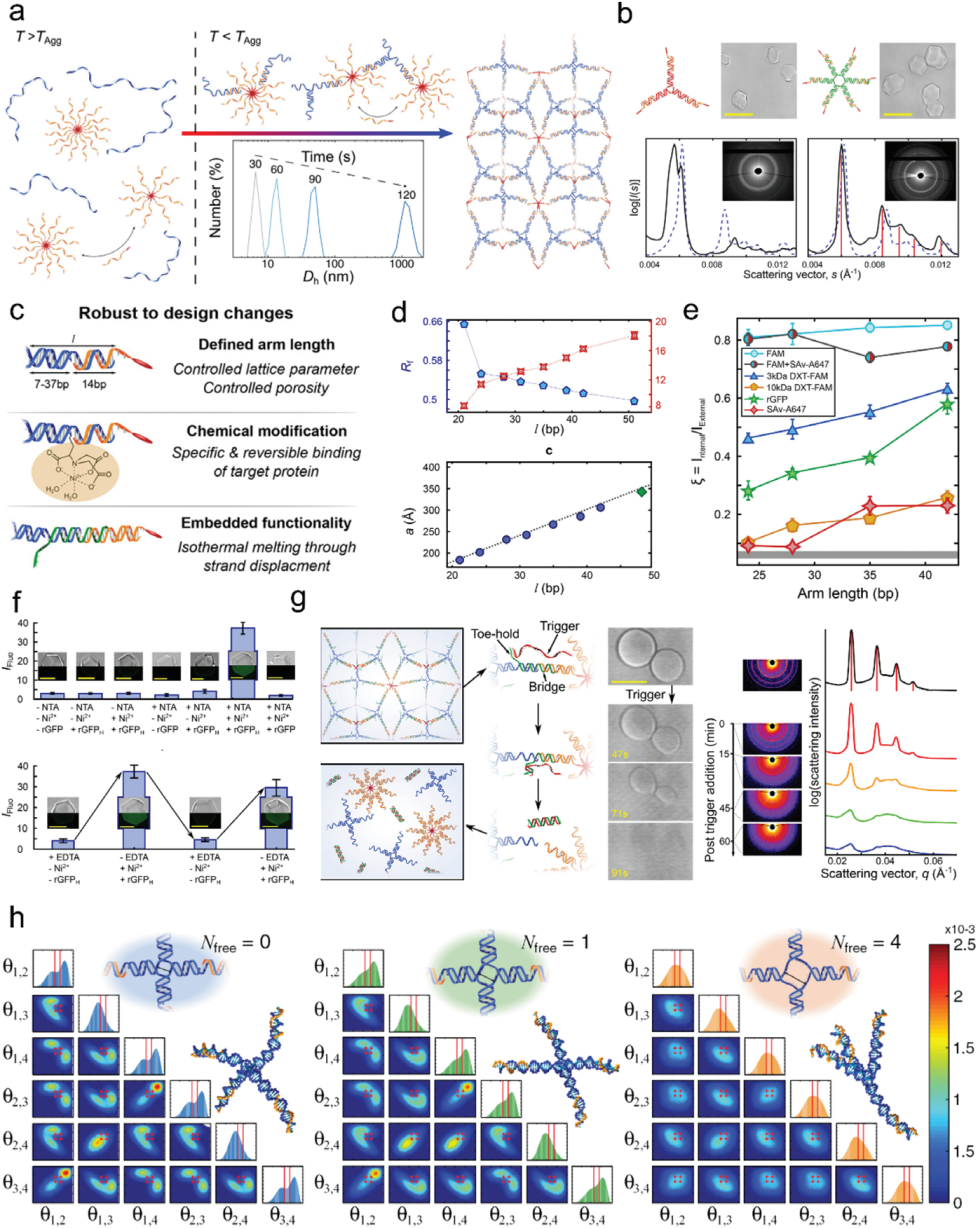
Amphiphilic DNA C‐star crystals. a) Slow cooling self‐assembly process of amphiphilic DNA C‐star crystals. T>TAgg: micelles coexist with single‐stranded core strands; T<TAgg: C‐Star cores bridge together existing micelles, promoting nucleation and crystallization. b) The secondary structure, crystal images, SAXS powder diffraction patterns, and radial intensity profiles for *n* = 3 and *n* = 6 amphiphilic DNA C‐star crystals. Reproduced with permission.^[^ ^15^ ^]^ Copyright 2017, American Chemical Society. c) Multifunctional amphiphilic DNA C‐star crystal platform with defined arm length, chemical modification and embedded functionality. d) The relative electrophoretic migration distance Rf decreases linearly with l in the range of 24−51 bp. The measured lattice parameter increases linearly with l in the range of 24−51 bp. e) The ratio *ξ* between the mean fluorescence levels inside and outside individual crystals soaked in solutions containing fluorescent probes. The porosity of amphiphilic DNA C‐star crystals could be controlled by adjusting arm length. f) Specific and reversible entrapment of poly histidine‐tagged rGFP (rGFPH) by modulating NTA modification, NiCl_2,_ and EDTA. g) Rapid isothermal melting of amphiphilic DNA C‐star crystals. Reproduced with permission.^[^ ^49^ ^]^ Copyright 2018, American Chemical Society. h) Conformational flexibility of individual DNA C‐stars with N_free_ = 0,1,4, investigated by coarse‐grained simulations. The flexibility increased with N_free_. Reproduced with permission.^[^ ^50^ ^]^ Copyright 2019, IOP Publishing.

The formation of the above‐mentioned DNA crystals requires precise design and reliable self‐assembly of DNA motifs with high structural rigidity and accurate bond orientation, which is highly challenging. To mitigate the complexity of the process, Di Michele et al. designed the amphiphilic DNA C‐star structure which consisted of four flexibly linked double strands paired with four cholesterol‐modified complementary strands. The assembly process of amphiphilic DNA C‐star crystal was investigated (**Figure** **7**a). In the absence of core strands, the cholesterol‐modified strands formed micelles with a hydrodynamic diameter of 8.5 ± 1.5 nm. At high temperatures (T > TAgg = 77.1 ± 0.2 °C), cholesterol‐DNA micelles coexist with the free core strand. During slow quenching process, phase transitions occurred as the temperature crossed TAgg. Self‐assembly of the core strands in the C‐star mediated by Watson–Crick base pairing triggered polymerization, which linked existing micelles together and led to rapid nucleation and crystallization. In contrast to conventional all‐DNA motifs, amphiphilic C‐stars have no fixed binding valence as their nucleation is supported by hydrophobic modifications. Flexible DNA motifs controlled the connected arrangement of micelles in space, ultimately determining the crystal structure. They also demonstrated that C‐stars consisting of three or six core strands could likewise form crystals (Figure 7b).^[^ ^15^ ^]^ In this case, it is no longer necessary to strictly control the 3D geometry of the DNA motif, thus lifting the design constraints that have always prevented the diversification of all‐DNA crystals.

In 2018, Brady et al. developed the amphiphilic DNA C‐star into a multifunctional crystal platform (Figure 7c).^[^ ^49^ ^]^ By adjusting the arm length of the core strands, the lattice size could be tuned. When the arm length was extended from 21 bp to 51 bp, the lattice size increased linearly, in high accordance with theoretical fitting values (Figure 7b). When the arm length was 51 bp, crystals did not form, possibly due to the increased flexibility of the longer DNA arms, which were too flexible to maintain a rigid network. They chose a variety of fluorescent probes covering a wide range of molecular weights, hydrodynamic sizes, hydrophobicity, and chemical properties, including sodium fluorescein (FAM), fluorescein‐modified dextrans (3 kDa DXT‐FAM, 10 kDa DXT‐FAM), rhodamine B, recombinant green fluorescent protein (rGFP) and fluorescently labeled streptavidin (SAvA647). The penetration of these fluorescent molecules in the amphiphilic DNA C‐star crystal was investigated and results showed that as the arm length increased, larger fluorescent molecules could be accommodated to penetrate into the interior of the crystal. Interestingly, rhodamine B penetrated efficiently into the crystal regardless of arm length, possibly due to the affinity for the cholesterol‐rich cores (Figure 7c,d,e). A functional group (nitrilotriacetic acid, NTA) was used to modify the core strands of amphiphilic DNA C‐star crystals, which were soaked with his‐tagged GFP (Figure 7f). GFP could enter the crystals without affecting the lattice parameters of the crystals. Finally, the isothermal dissolution of these amphiphilic DNA C‐star crystals was induced using a chain replacement reaction (Figure 7g). This multifunctional crystal platform had the advantage of structural responsiveness and programmability, as well as the specific and non‐specific affinity for various molecules. In 2019, Brady combined molecular dynamics simulations and small‐angle X‐ray scattering to investigate the role of structural rigidity in the crystallization of amphiphilic DNA C‐stars (Figure 7h),^[^ ^50^ ^]^ They investigated the effect of the number of unpaired bases in the core chains (Nfree = 0,1,4) and the concentration of sodium and magnesium ions in the buffer on crystal formation. The results showed that Nfree = 4 was most favorable for crystal formation, regardless of the concentrations of sodium and magnesium ions. In contrast, Nfree = 0 or 1 did not allow crystalizing in the presence of magnesium ions, probably due to the magnesium ions leading to junctions with rigid X‐shaped geometry. This study demonstrated how flexibility was not only an acceptable feature but a key factor in the successful crystallization of amphiphilic DNA C‐stars. This situation was in complete contrast to all‐DNA motifs that crystalized through Warson‐Crick base pairing or base stacking interactions, implying the possibility of designing more flexible DNA motifs with well‐defined topologies for crystallization.

## 6 Crystallization Conditions

For the crystallization of conventional biomolecules, such as proteins, it is usually necessary to perform protein expression, purification, and further error screening. Crystals of satisfactory sizes, shapes, and resolutions would be eventually obtained by adjusting temperatures, concentrations, buffer conditions and other factors during crystal growth. With the development of low‐cost DNA synthesis techniques, it has been relatively inexpensive to customize and purify oligonucleotide strands. Due to the features of Warson–Crick base pairing of DNA, key parameters for the assembly of 3D DNA crystals such as the temperature, ionic strength and crystallization method, will be described in turn below.

### 6.1 Temperature

**Figure 8:**
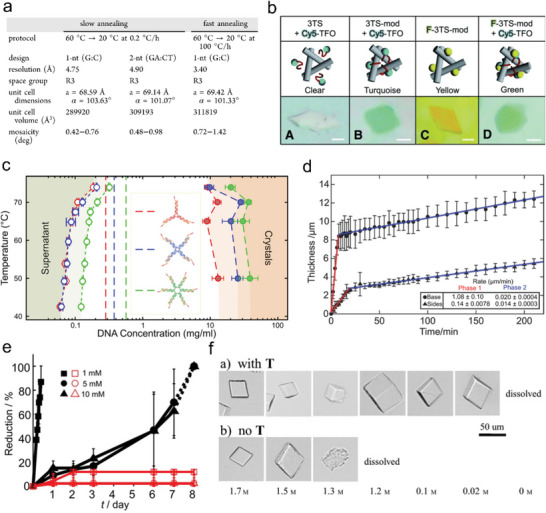
Crystallization conditions of 3D DNA crystals. a) Comparison of the X‐ray data of tensegrity triangle crystals obtained from slow and fast annealing procedures. Reproduced with permission.^[^ ^39^ ^]^ Copyright 2019, American Chemical Society. b) Fluorescein‐labeled crystals obtained in the presence of TFO. Reproduced with permission.^[^ ^34^ ^]^ Copyright 2014, Wiley‐VCH. c) Phase behavior of the amphiphilic DNA C‐star crystals. C‐Stars display a wide gas‐crystal coexistence region, reflecting the strong interactions driving aggregation and the high valency. Reproduced with permission.^[^ ^15^ ^]^ Copyright 2017, American Chemical Society. d) Time‐resolved growth kinetics of shell thickness on the base and the sides of the DNA 13‐mer crystals. The crystal showed biphasic linear accumulation over the time period. Reproduced with permission.^[^ ^19^ ^]^ Copyright 2017, Wiley‐VCH. e) Alkyl chain crosslinking decreases the Mg^2+^ concentration requirements of the DNA 13‐mer crystals. Reproduced with permission.^[^ ^18^ ^]^ Copyright 2016, Wiley‐VCH. f) The introduction of TFO reduced the concentration of ammonium sulfate in the tensegrity triangle crystal stabilization solution from 1.2 M to 0.02 M. Reproduced with permission.^[^ ^35^ ^]^ Copyright 2015, Wiley‐VCH.

Annealing is usually required in programmable DNA base pairing. The process of annealing involves high temperatures to completely denature single‐stranded DNA and then cool down to allow complementary base pairing to ensure correct crystal assembly. For DNA crystals based strictly on base pairing, such as the tensegrity triangle crystals and 4 × 6 motif crystals, a reduction in temperature from 60 °C to room temperature (20—25 °C) over a number of days at a cooling rate of 0.2–0.3 °C h^−1^ is required. During the cooling period, the droplet volume was reduced by approximately 90% to produce the crystals. Ohayon et al. studied the effect of rapid annealing (60–20 °C, 100 °C h^−1^) on the formation of the tensegrity triangle crystals and found that the diffraction resolution of the tensegrity triangle crystals was slightly improved, but the mosaicism was significantly enhanced (**Figure** **8**a).^[^ ^39^ ^]^ With the introduction of TFO, annealing conditions for the tensegrity triangle crystals were changed somewhat from the previous one‐step method to a two‐step method: first annealing to form the motif (90 °C for 5 min, 65 °C for 20 min, 45 °C for 20 min, 37 °C for 30 min and 20 °C for 30 min), followed by crystallization (22 °C or 4 °C, 2–7 days) (Figure 8b).^[^ ^34^ ^]^ This approach greatly reduced the annealing time and facilitated various post‐modifications and accommodation of guest molecules. For the amphiphilic DNA C‐star crystals, it needs to be held at 95 °C for 30 min and cooled down to 30 °C at −0.05 °C min^−1^ to −0.005 °C min^−1^ (Figure 8c).^[^ ^15^ ^]^ A gel‐like substance will be produced when the annealing rate increases, which was determined by the assembly properties of the cholesterol hydrophobic groups. The DNA 13‐mer crystal, on the other hand, was mainly formed by non‐base complementary pairing and can therefore be crystallized at a lower temperature (37 °C overnight, cooled down to 22 °C; or grown at a constant temperature of 22 °C) (Figure 8d).^[^ ^19^ ^]^

### 6.2 Ions

DNA 13‐mer crystals, tensegrity triangle crystals, and 4 × 6 motif crystals required higher concentrations of magnesium ions (12.5–50 mM) and high ionic strength to stabilize the DNA motifs and interactions between DNA motifs. Naturally, such a solution environment is also unfriendly to most guest molecules. Zhang et al. reduced the concentration of magnesium ions in the DNA 13‐mer crystal stabilization buffer from 40 mM to 1 mM by cross‐linking alkyl chains (Figure 8e).^[^ ^18^ ^]^ Seeman et al. reduced the concentration of ammonium sulfate in the tensegrity triangle crystal stabilization solution from 1.2 M to 0.02 M by the introduction of TFO (Figure 8f).^[^ ^35^ ^]^ Amphiphilic DNA C‐star motifs required a certain degree of flexibility and therefore did not require the divalent ions normally added at high concentrations to stabilize the DNA structure. The ionic strength required for crystallization was much lower and crystallization could usually be achieved in Tris‐EDTA buffer containing an additional 300 mM NaCl.

### 6.3 Crystallization Methods

Conventional crystallization methods of biological macromolecules usually include the sitting‐drop method and the suspension‐drop method based on vapor diffusion. For the DNA 13‐mer crystals, tensegrity triangle crystals and 4 × 6 motif, the sitting‐drop method is commonly used, utilizing the diffusion of solvent vapor from low to high concentrations to gradually increase the concentration of the DNA motifs and thus produce crystals.^[^ ^12^, ^13^, ^14^ ^]^ The crystallization of amphiphilic DNA C‐star is completely different as it does not depend on the increase in the concentration of the DNA structure in the system and is entirely cholesterol‐mediated self‐assembly. Single‐stranded DNA is heated to 95 °C for complete denaturation, then loaded into a completely cleaned capillary tube sealed on both sides with mineral oil, followed by a slow annealing procedure.^[^ ^15^ ^]^

## 7 Perspectives

Nucleic acid crystallography originated from the study of DNA double helix. Franklin and Gosling collected diffraction images of microcrystals of DNA fibers,^[^ ^51^ ^]^ which allowed Crick and Watson to model the repeating structure of the double‐stranded DNA helix, revealing the beautiful and striking three‐dimensional architectures of DNA.^[^ ^52^ ^]^ In 1973, the first single‐crystal structure of a nucleic acid (tRNA) was resolved.^[^ ^53^ ^]^ With the rapid development of DNA synthesis and purification techniques, DNA with specific sequences could be customized optionally. In 1980, the first all‐base‐paired B‐DNA (CGCGAATTCGCG) structure was determined by single‐crystal methods with a resolution of 1.9 Å.^[^ ^54^ ^]^ In 1999, the first Holiday junction structure (CCGGGACCGG) was crystallized and resolved.^[^ ^55^ ^]^

In 1982, Seeman proposed that DNA could be used as a programmable structural material for rationally designed branching junctions based on Warson–Crick base pairing.^[^ ^8^ ^]^ This in turn led to the use of DNA as a base module for the construction of 3D DNA structures, establishing the basis for DNA nanotechnology. Initially, the assembly of DNA molecules into designed periodic arrays (i.e., crystals) that act as porous scaffolds to orient and position guest molecules (e.g., proteins) in specific locations, overcome bottlenecks in macromolecular crystallization and enable rapid structure determination was an important goal of DNA nanotechnology. Artificially designed nanoparticle crystals are important for understanding the crystallization processes, rationally controlling material properties, and building smart devices.^[^ ^56^ ^]^ In 1996, Mirkin et al. first suggested that DNA could act as a programmable bond between colloidal nanoparticles to assemble nanoparticles into discrete, well‐defined, homogeneous, and soluble macroscopic aggregates.^[^ ^57^, ^58^ ^]^ Their demonstration opened the field of crystallization of nanoparticles engineering with DNA. Over the next two decades, DNA nanotechnology offered the ability to introduce highly addressable bonds, tailor particle interactions, and control the geometry of bound motifs. Mirkin, et al. had used rationally designed single‐stranded DNA and DNA origami as linking bonds to construct a series of nanoparticle crystals, and made much work in the study of the mechanisms, rules, regulation, dynamics, and applications of nanoparticles crystals.^[^ ^59^, ^60^, ^61^, ^62^, ^63^, ^64^, ^65^, ^66^, ^67^ ^]^ In the future, nanoparticles crystals engineering with DNA are expected to have broad implications in energy materials, information storage, electronics, optics, and catalysis.

Currently, DNA nanotechnology has moved in many directions and grown beyond the original envisions of Seeman. DNA origami technology is in full swing, allowing the creation of a wide range of programmable two‐ and 3D DNA structures, by folding a long ssDNA as designed using hundreds of “staples” made of short complementary ssDNA.^[^ ^68^, ^69^, ^70^ ^]^ However, there are still significant differences between DNA origami structures and 3D DNA crystals, including spatial heterogeneity, structural defects, and product purification, which prevent them from being used as guest molecular scaffolds to resolve the guest molecule structure.^[^ ^11^, ^70^ ^]^ Designing and assembling origami units into ordered superlattices or 3D crystals was very challenging due to the size of DNA origami and the complicated interactions between origami structures. In recent years, a series of works had been carried out to construct 3D DNA crystals using designed larger origami building blocks. In 2018, Liedl's group replicated the tensegrity triangle geometry with origami to construct DNA origami crystal. Based on the stacking interactions, they assembled DNA‐origami‐based “tensegrity triangle” structure into a 3D rhombohedral lattice that can efficiently accommodate 20 nm nanoparticles in a cavity size of 1.83 × 10^5^ nm^3^, which was also large enough to accommodate ribosome‐sized guest molecules. The lattice was almost invisible under x‐ray and electron irradiation and thus had great potential for structural resolution of incorporated guest molecules.^[^ ^71^ ^]^ In 2020, Gang's group assembled octahedral DNA origami into a 3D DNA superlattice with 48 nm unit cell. By mineralizing this 3D DNA superlattice to form a highly structured 3D silicon scaffold and further coating with superconducting niobium (Nb), a superconductive superlattice was constructed. This strategy could potentially be used to fabricate arbitrarily designed 3D superconducting materials with precise nano‐ and mesoscopic organization.^[^ ^72^ ^]^ Gang's group also assembled octahedral, cubic, and tetrahedra DNA origami into 3D DNA superlattices. Quantum dots and enzymes were introduced into 3D DNA superlattices to generate optical functional and enzymatic cascade reactions.^[^ ^73^ ^]^ Tian's group constructed dynamically reconfigurable DNA origami crystals with multiphase transformation capabilities, rigid DNA rods‐enhanced DNA origami crystals, DNA origami single crystals with Woolf shapes.^[^ ^70^, ^74^, ^75^, ^76^ ^]^ DNA origami crystals have a large lattice completely determined by the origami monomer design, which allows for precise design of the lattice and further regulation of the positioning of the guest molecule, therefore have a unique advantage in serving as a vehicle for the structural resolution of the guest molecules.

At present, there are still very few fully designed 3D DNA crystals, mainly because the design, crystallization and structural resolution of DNA crystals require deep knowledge and understanding of the DNA nanostructure as well as rigorous training in crystallography. Of course, with the rapid development of DNA nanotechnology and increased collaboration between many research groups, increasing numbers of 3D DNA crystals are expected to be developed. Despite the current efforts of researchers, there has been a qualitative leap in cavity size, solvent content, diffraction resolution, and ability to accommodate guest molecules in existing 3D DNA crystals. However, there are still no reports of 3D DNA crystals being used to resolve the structures of guest molecules. Even after accommodating the guest molecules, no relevant diffraction patterns have been detected. This is mainly due to the fact that the guest molecules exist in many different orientations in the solvent channels. It is therefore important to use well‐defined environments for the guest molecules in the 3D DNA crystals. Furthermore, the inherent negative charge of nucleic acids, the high solvent content and varying structural dynamics pose many challenges to the formation of highly ordered 3D DNA crystals. Recently, Zhang et al. demonstrated a new design principle to engineer precisely ordered, symmetry‐tunable DNA crystals that diffract to 2.2 Å. The high resolution of engineered crystals enabled study of the structural details of DNA−guest molecule interactions. The guest molecule incorporated in the DNA crystals could be unambiguously modeled into the electron density map. This study achieved an important advancement that resolving the structure of guest molecules by 3D DNA crystals.^[^ ^77^ ^]^

In 2016, Boerneke et al. rationally designed two different RNA nanotriangles self‐assembled from six oligonucleotides.^[^ ^78^ ^]^ The RNA nanotriangles were crystalized and the crystal structure of the RNA nanotriangles was revealed at 2.6 Å resolution. By encoding ligand‐responsive RNA switches into the motifs, 3D RNA crystals are expected to be developed as tunable environmentally responsive machines. In 2018, Weizmann et al. construction of RNA nanostructures based on homo‐oligomerizable one‐stranded tiles, and resolved the crystal structures of three synthetic RNA nanoarchitectures. They demonstrated the feasibility of getting crystal structures from discrete RNA nanostructures and to elucidate the structural details.^[^ ^79^ ^]^ At present, structural resolution of 3D DNA crystals usually relied on multiwavelength anomalous dispersion (MAD) phasing based on bromine derivatization of nucleic acids (BrNA). The selenium derivatization of nucleic acids (SeNA) developed by Huang et al. is more stable than BrNA and is more conducive to DNA crystallization and structural resolution. Thus, SeNA technology is expected to be a favorable tool to facilitate 3D DNA crystals.^[^ ^80^, ^81^ ^]^ Currently, the rapidly expanding synchrotron radiation X‐ray crystallography beamlines offer effective resources for the investigation of 3D DNA crystals. As an illustration, currently, there are 7 beamlines available at the Shanghai Synchrotron Radiation Facility for collecting crystal diffraction data, offering X‐rays with various light intensities, spot sizes, and divergence angles and experimental environments, which can meet a variety of experimental needs, including (BSL‐2 biological protection, biological macromolecular complexes, membrane proteins, etc.).^[^ ^82^, ^83^, ^84^, ^85^, ^86^ ^]^

As the toolbox of DNA nanotechnology continues to evolve and diversify, we also expect the novel advances of 3D DNA crystals in other fields: 1) The potential of 3D DNA crystals as solid‐state modular catalysts can be exploited by introducing different enzymes into the crystal units or solvent channels ^[^ ^23^, ^87^, ^88^ ^]^; 2) the 3D DNA crystals could act as biosensors by incorporation of molecular recognition elements (enzymes, antigens, antibodies, aptamers, etc.) and corresponding transducers ^[^ ^3^, ^89^ ^]^; 3) by designing the unit and solvent channels in 3D DNA crystals to enhance the localization accuracy of the guest molecules in 3D DNA crystals, the high‐precision molecular sieve with the potential to screen, capture and release specific macromolecules in complex systems could be achieved ^[^ ^21^, ^90^ ^]^; 4) organic semiconductor molecules or electroactive compounds could be organized in 3D DNA crystals, to construct complex and intelligent electronic systems such as nano‐electronic components, photovoltaic devices, and semiconductor sensors by designing their positioning, density, and orientation ^[^ ^43^, ^91^ ^]^; 5) 3D DNA crystals have been shown the features of reversibly expand/contract and sequence‐responsive melting. After functionalization with ligands or DNA aptamers, 3D DNA crystals could serve as drug carriers to achieve selective targeting of macromolecule drugs (e.g., nucleic acids and proteins) to specific cells/organ, which of course cannot be achieved without further enhancements of the biocompatibility and stability of DNA crystals within biological systems ^[^ ^44^ ^]^; 6) After a lot of efforts, we may be able to achieve the modular design of 3D DNA crystals based on coupling of DNA molecular reaction networks with enzymatic processes, and further complete the construction of highly ordered DNA‐based synthetic cells with energy conversion, communication, motion, and adaptation functions.^[^ ^92^, ^93^ ^]^

Seeman, who founded the area of structural DNA nanotechnology, has contributed most to its advancement and generated so much interest that it has grown into a separate discipline. There are now more than 250 labs worldwide working in the field of structural DNA nanotechnology. With the continuous development of DNA nanotechnology and the growth of young scientists, it will not be surprising at all to see more exciting innovations in coming decades within the field of DNA crystal and DNA nanotechnology.

## Conflict of Interest

The authors declare no conflict of interest.
